# Post‐endoscopic fever and infection in paediatric patients with intestinal failure

**DOI:** 10.1002/jpn3.70141

**Published:** 2025-07-03

**Authors:** Johannes Hilberath, Omar Afrigh, Toni Illhardt, Drieke Vermeulen, Christoph Slavetinsky, Tobias Jhala, Bernd Fode, Hanna Renk, Justus Lieber, Jörg Fuchs, Ekkehard Sturm

**Affiliations:** ^1^ Paediatric Gastroenterology and Hepatology, Department of Haematology and Oncology University Children's Hospital Tübingen Tübingen Germany; ^2^ Paediatric Surgery and Urology University Children's Hospital Tübingen Tübingen Germany; ^3^ General Paediatric, Department of Haematology and Oncology University Children's Hospital Tübingen Tübingen Germany; ^4^ Institute of Medical Microbiology and Hygiene University Hospital Tübingen Tübingen Germany

**Keywords:** antimicrobial prophylaxis, central line‐associated bloodstream infection, paediatric gastrointestinal endoscopy, transient bacteraemia

## Abstract

**Objectives:**

Routine antimicrobial prophylaxis (AMP) for preventing bacteraemia and infection during paediatric gastrointestinal (GI) endoscopy is not recommended and is reserved for high‐risk scenarios. However, in the unique group of children with intestinal failure (IF) and a central venous catheter (CVC), the incidence of post‐endoscopic fever (PEF) and infection and the usefulness of AMP in protecting the indwelling catheter are unknown. This study evaluated fever and infection rates post‐endoscopy, and the role of AMP in children with IF and CVC.

**Methods:**

This retrospective single‐centre observational study included children with IF and CVC who underwent GI endoscopy at our intestinal rehabilitation centre between 2019 and 2024. Owing to a policy change, routine AMP was terminated in 2022. AMP group (intravenous [i.v.] antibiotics) and no‐AMP group (no i.v. AMP) were compared using chi‐square and Mann–Whitney *U* tests.

**Results:**

A total of 233 endoscopies in 108 in‐patients with IF and CVC were analysed: median age at endoscopy, 68 months (range: 1–206 months); female, 54.6%; short bowel syndrome, 73.1%. Intravenous AMP was used in 71.2% of the procedures. Median follow‐up after endoscopy was 2 days. There were no differences between the AMP and no‐AMP groups in terms of age, type of endoscopy, interventional procedures, or pre‐endoscopic use of enteral antibiotics or proton‐pump inhibitors. The overall PEF rate was 6%, with no significant difference between groups. No infections, including central line‐associated bloodstream infections, were observed.

**Conclusions:**

The frequency of PEF in children with IF is approximately 10 times higher than the recently reported incidence rate of 0.55% in paediatric patients. Since no bloodstream infections were confirmed, and AMP did not prevent PEF, routine administration of AMP for diagnostic endoscopy in children with IF is not indicated.

## Abstract (highlights)

**What is Known:**

- Bacteraemia can occur during gastrointestinal (GI) endoscopy; however, the incidence of post‐endoscopic fever (PEF) and infection in children with intestinal failure (IF) are unknown.
- Routine antimicrobial prophylaxis (AMP) during paediatric GI endoscopy to prevent bacteraemia and infection is not recommended and is reserved for high‐risk scenarios.
- The usefulness of intravenous AMP in children with IF and a central venous catheter (CVC) is unknown, and clinical practice is likely to vary across centres.

**What is New:**

- PEF was a frequent finding in paediatric patients with IF; however, no infections, including central line‐associated bloodstream infections occurred.
- Routine AMP does not prevent PEF or infection in children with IF and CVC.
- These findings may have implications for clinical practice and guideline development.

## Graphical abstract

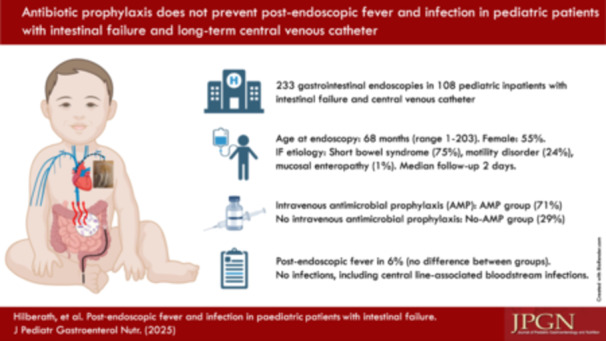

## 1 INTRODUCTION

Children with intestinal failure (IF) depend on long‐term central venous catheters (CVC) to provide home parenteral nutrition. However, infectious complications, including central line‐associated bloodstream infections (CLABSIs), account for relevant morbidity and mortality, and may eventually necessitate an evaluation of intestinal transplant.^1^, ^2^, ^3^ Gastrointestinal (GI) endoscopy is a common procedure in this unique group of patients, as it has a high diagnostic yield and a relevant impact on management.^4^, ^5^ However, endoscopy‐associated endogenous bacteraemia and infections have been reported, and despite considering children with IF as high‐risk patients, there are no published data on infective adverse events.^6^, ^7^ While in an earlier publication from 2002, 20%–40% of paediatric centres reported to perform antimicrobial prophylaxis (AMP) for GI endoscopic procedures in children with a CVC,^8^ current data on real‐life management in this patient group is not available, and practice is likely to vary across centres. However, it is unclear whether preventive antibiotic treatment is required and whether effective control of bacteraemia can be expected.

Therefore, the aims of this study were (1) to investigate the frequency of post‐endoscopic fever (PEF) and infection rate in children with IF and CVC and (2) to compare these outcomes in children receiving pre‐endoscopic AMP versus no‐AMP. The findings of this study may have implications for clinical practice and guideline development in this vulnerable patient population.

## 2 METHODS

This was a retrospective, observational, single‐centre study of all children (0–17 years) with IF and CVC who underwent GI endoscopy at our intestinal rehabilitation centre between 2019 and 2024. Endoscopies were performed either as screening procedures as part of the work‐up in patients with IF or in cases with GI dysfunction. The University Children's Hospital Tübingen, Germany, is a national referral centre for paediatric intestinal rehabilitation and transplantation. Outpatients were excluded from this study.

Clinical, diagnostic and management data were collected from medical records: patient characteristics including underlying IF aetiology and medication before endoscopy, indication and type of endoscopy, provision of AMP, and occurrence of fever or infection within 24 h post‐endoscopy. Standard monitoring after GI endoscopy included the recording of vital parameters such as body temperature at least once per shift. Fever was defined as a patient's temperature ≥38.0°C and was measured by health care professionals during hospital admission. All endoscopies were performed by an endoscopist trained in paediatric gastroenterology.

In accordance with our IF centre's concept of vascular rehabilitation,^9^ patients' central venous lines were solely for providing parenteral nutrition and in case of emergencies. In this regard, CVCs were not used for blood sampling or the administration of fluids or drugs, including anaesthetic procedures for GI endoscopy.

Owing to a policy change in 2022, routine AMP with intravenous (i.v.) piperacillin/tazobactam (200 mg piperacillin component/kg/day in three divided doses starting the day before endoscopy and continued for 3–5 days) was stopped at our centre. To determine the frequency of PEF and infection in relation to administered or non‐administered AMP, two groups were defined: the AMP group (i.v.‐antibiotics) and the no‐AMP group (no i.v.‐AMP).

### 2.1 Ethics statement

The study was conducted according to the ethical principles of the 1975 Declaration of Helsinki and approved by the Ethics Committee at the Medical Faculty of the Eberhard Karls University and at the University Hospital of Tübingen (30 October 2023; reference 606/2023BO2). As decided by the Ethics Committee, informed consent was obtained from patients/caregivers who could be approached with reasonable effort.

### 2.2 Statistics

Descriptive analysis was performed using IBM® SPSS® Statistics, version 28.0, and the groups were compared using the chi‐square test for two categorical variables or the Mann‐Whitney U‐Test; a *p*‐value < 0.05 was considered statistically significant.

## 3 RESULTS

Between 2019 and 2024, a total of 233 endoscopies in 108 in‐patients with IF and parenteral nutrition dependency via a long‐term CVC were performed: median age at endoscopy, 68 months (range: 1–206 months); female patients, 54.6% (*n* = 59); short bowel syndrome, 75.1% (*n* = 175); motility disorder 23.6% (*n* = 55); mucosal enteropathy 1.3% (*n* = 3). The median follow‐up time until discharge after endoscopy was 2 days (interquartile range: 1–4). None of the patients were immunocompromised.

**Figure 1:**
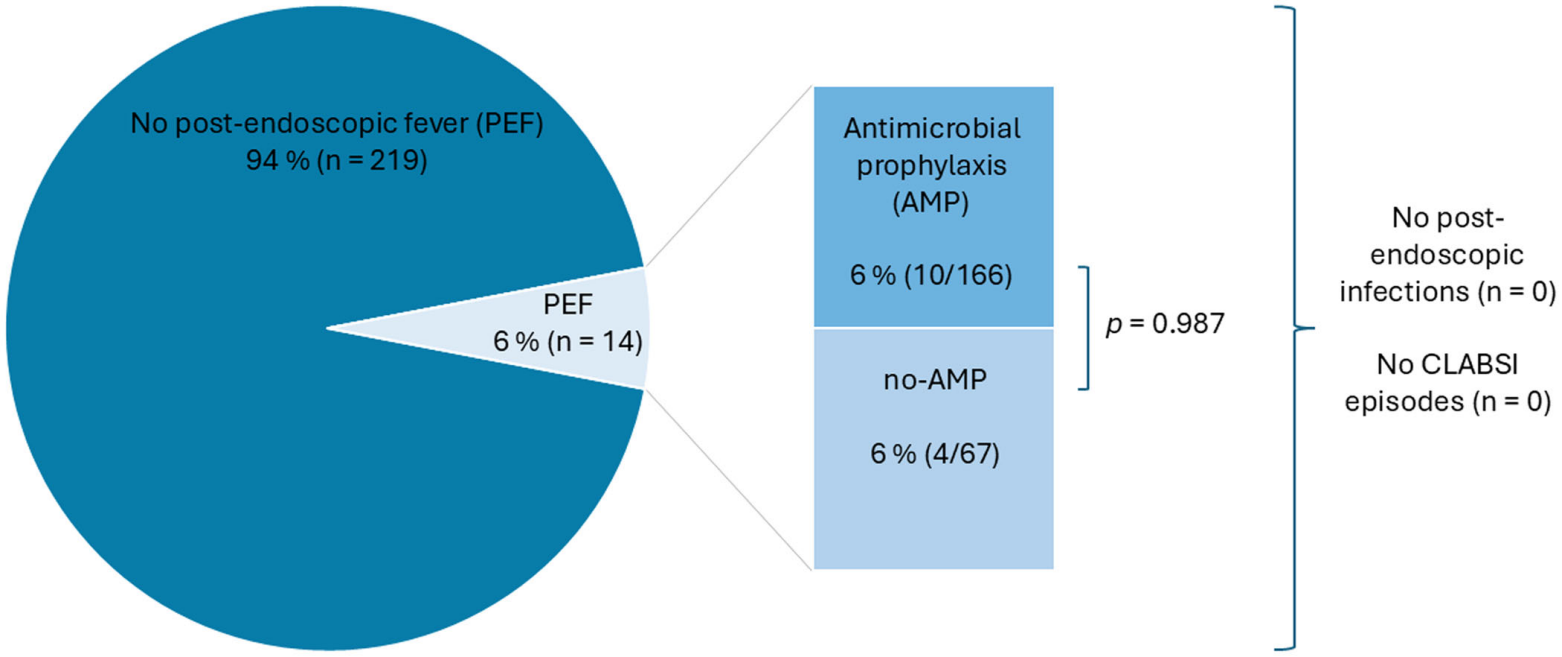
Frequency of PEF/infection after 233 endoscopies in 108 in‐patients with intestinal failure and central venous catheter and comparison between the AMP and no‐AMP groups. AMP, antimicrobial prophylaxis; CLABSI, central line‐associated bloodstream infection; PEF, post‐endoscopic fever. Chi‐square test for two categorical variables.

Intravenous AMP was used in 71.2% of the procedures. In 16 cases (9.6%), an alternative to piperacillin/tazobactam as i.v. antibiotic regime was provided (meropenem 6×; meropenem/vancomycin 1×; meropenem/ampicillin/sulbactam 1×; ciprofloxacin 3×; cefotaxime 1×; cefotaxime/ampicillin 1×; cefotaxime/tobramycin 1×; cefotaxime/metronidazole 1×; and vancomycin/cefepime 1×). The reason was documented in five cases as a known allergy to piperacillin. There were no statistically significant differences between the AMP and no‐AMP groups in terms of age, type of endoscopy, interventional procedures, or pre‐endoscopic use of proton pump inhibitors (PPIs) or enteral antibiotics (Table 1). The overall rate of PEF was 6% (*n* = 14), with no significant differences between the groups (Figure 1). Among the patients with PEF, C‐reactive protein levels were assessed in half, revealing a slight increase in six children (with a peak of 3.9 mg/dL) and a normal level in one child. No infections, including CLABSIs, were observed. Peripheral blood cultures were analysed in two patients with PEF (14.3%), revealing negative results. PEF was self‐limiting in all cases, and no intervention was necessary. However, the patient was only discharged from the hospital after the resolution of fever.

**Table 1:** Characteristics of endoscopic procedures and comparison between the AMP group (pre‐endoscopic i.v. antibiotic treatment) and the no‐AMP group (no i.v.‐antibiotics).

	Total	AMP group	No‐AMP group	*p*
GI endoscopies (*n*, %)	233	166/233 (71.2%)	67/233 (28.8%)	
EGD (*n*, %)	221 (94.8%)	156/166 (94.0%)	63/67 (94%)	0.935
Colonoscopy (*n*, %)	142 (60.9%)	102/166 (61.4%)	38/67 (56.7%)	0.515
Stomascopy (*n*, %)	34 (14.6%)	26/166 (15.7%)	8/67 (11.9%)	0.407
Age at endoscopy [months, median (range)]	68 (1–206)	63 (1–203)	73.5 (2–206)	0.337
Pre‐endoscopic oral or enteral antibiotics	37/233 (15.9%)	31/166 (18.7%)	6/67 (9%)	0.066
Pre‐endoscopic PPI therapy	104/233 (44.6%)	71/166 (42.8%)	33/67 (49.3%)	0.368
Mucosal biopsies (*n*, %)	178/233 (76.4%)	125/166 (75.3%)	50/67 (74.6%)	0.914
Interventional procedures				
‐ PEG placement (*n*, %)	16/233 (6.9%)	14/166 (8.4%)	2/67 (3%)	0.137
‐ Tube placement/change (*n*, %)	49/233 (21%)	30/166 (18.1%)	19/67 (28.4%)	0.081
‐ Variceal banding (*n*, %)	6/233 (2.6%)	4/166 (2.4%)	2/67 (3%)	0.802
‐ ERCP (*n*, %)	2/233 (0.9%)	2/166 (1.2%)	0/67 (0%)	0.367
‐ Intestinal stent placement (*n*, %)	2/233 (0.9%)	1/166 (0.6%)	1/67 (1.5%)	0.505
‐ Local ink injection (*n*, %)	2/233 (0.9%)	2/167 (1.2%)	0/67 (0%)	0.367

*Note*: Chi‐square test for two categorical variables or Mann–Whitney *U* test.

Abbreviations: AMP, antimicrobial prophylaxis; EGD, esophagogastroduodenoscopy; ERCP, endoscopic retrograde cholangiopancreatography; GI, gastrointestinal; i.v., intravenous; PEG, percutaneous endoscopic gastrostomy; PPI, proton pump inhibitor.

Patient's and endoscopic characteristics of the 14 cases with PEF are shown in Table 2. Subgroup analysis of all PEF cases in relation to administered or non‐administered AMP showed no statistically significant differences (Table 3).

**Table 2:** Characteristics of the 14 cases with post‐endoscopy fever.

#	Underlying disease	Age (months)	Gender	Enteral antibiotic	PPI	Type of endoscopy	Mucosal biopsy	Endoscopic intervention
*Intravenous AMP*								
1	SBS	59	M	No	No	EGD + Colo	Yes	No
2	SBS	49	F	No	Yes	EGD	No	Yes (variceal banding)
3	SBS	44	F	No	No	EGD + Colo	Yes	No
4	SBS	49	F	No	Yes	EGD + Colo	Yes	No
5	MD	98	F	No	No	EGD + Colo	Yes	No
6	SBS	30	M	No	No	EGD + Colo	No	No
7	SBS	99	F	Yes	Yes	EGD	No	Yes (ink injection)
8	MD	157	F	No	Yes	EGD	Yes	Yes (tube placement)
9	SBS	120	M	Yes	Yes	EGD	No	No
10	SBS	35	F	No	Yes	Colo	No	Yes (tube placement)
*No intravenous AMP*								
11	SBS	163	F	No	No	EGD + Colo	Yes	No
12	SBS	42	M	No	No	EGD + Colo	Yes	No
13	SBS	25	F	No	Yes	EGD + Colo	Yes	Yes (tube placement)
14	SBS	164	M	No	No	EGD + Colo	Yes	No

Abbreviations: AMP, antimicrobial prophylaxis; Colo, colonoscopy; EGD, esophagogastroduodenoscopy; F, female; M, male; MD, motility disorder; PPI, proton pump inhibitor; SBS, short bowel syndrome.

**Table 3:** Comparison of the 14 cases with PEF with and without intravenous AMP.

	Total	AMP group	No‐AMP group	*p*
Cases of PEF (*n*, %)	14	10	4	
Age at endoscopy [months, mean (range)]	81 (25–164)	74 (30–157)	98.5 (25–164)	0.839
Underlying IF aetiology				
‐ Short bowel syndrome	12/14 (85.7%)	8/10 (80%)	4/4 (100%)	0.334
‐ Motility disorder	2/14 (14.3%)	2/10 (20%)	0/4 (0%)	0.334
Female (*n*, %)	9/14 (64.3%)	7/10 (70%)	2/4 (50%)	0.480
Colonoscopy (*n*, %)	10/14 (71.4%)	6/10 (60%)	4/4 (100%)	0.134
Pre‐endoscopic oral/enteral antibiotics (*n*, %)	2/14 (14.3%)	2/10 (20%)	0/4 (0%)	0.334
Pre‐endoscopic PPI therapy (*n*, %)	7/14 (50.0%)	6/10 (60%)	1/4 (25%)	0.237
Mucosal biopsies (*n*, %)	9/14 (64.3%)	5/10 (50%)	4/4 (100%)	0.078
Interventional procedures	5/14 (35.7%)	4/10 (40%)	1/4 (25%)	0.597
‐ Tube placement/change (*n*, %)	3/14 (21.4%)	2/10 (20%)	1/4 (25%)	0.837
‐ Variceal banding (*n*, %)	1/14 (7.1%)	1/10 (10%)	0/4 (0%)	0.512
‐ Local ink injection (*n*, %)	1/14 (7.1%)	1/10 (10%)	0/4 (0%)	0.512

*Note*: Chi‐square test for two categorical variables or Mann–Whitney *U* test.

Abbreviations: AMP, antimicrobial prophylaxis; IF, intestinal failure; PEF, post‐endoscopic fever; PPI, proton pump inhibitor.

A comparison and analysis of all cases with and without PEF are presented in Table 4. Two patients underwent local ink injection to facilitate the surgical identification of the area of interest during laparotomy. One of these patients developed PEF despite receiving AMP, whereas the other patient did not (no‐AMP group).

**Table 4:** Comparison of all cases with and without PEF.

	PEF	No PEF	*p*
Endoscopies (*n*, %)	14/233 (6%)	219/233 (94%)	
Age at endoscopy [months, mean (range)]	81 (25–164)	77 (1–206)	0.734
Underlying IF aetiology			
‐ Short bowel syndrome (*n*, %)	12/14 (85.7%)	163/219 (74.4%)	0.344
‐ Motility disorder (*n*, %)	2/14 (14.3%)	53/219 (24.2%)	0.397
‐ Mucosal enteropathy (*n*, %)	0/14 (0%)	3/219 (1.4%)	0.659
Female (*n*, %)	9/14 (64.3%)	102/219 (46.6%)	0.198
Colonoscopy (*n*, %)	10/14 (71.4%)	132/219 (60.3%)	0.407
Pre‐endoscopic oral/enteral antibiotics (*n*, %)	2/14 (14.3%)	35/219 (16%)	0.866
Pre‐endoscopic PPI therapy (*n*, %)	7/14 (50.0%)	97/219 (44.3%)	0.677
Mucosal biopsies (*n*, %)	9/14 (64.3%)	164/219 (74.9%)	0.379
Interventional procedures	5/14 (35.7%)	72/219 (32.9%)	0.827

*Note*: Chi‐square test for two categorical variables or Mann–Whitney *U* test.

Abbreviations: IF, intestinal failure; PEF, post‐endoscopic fever; PPI, proton pump inhibitor.

## 4 DISCUSSION

This is the first study to investigate (1) the frequency of PEF/infection in paediatric patients with IF and (2) the role of AMP.

In our study, the rate of PEF in children with IF was 6%, which is approximately 10 times higher than the recently published 0.55% in paediatric patients following endoscopic procedures by Boster et al.^10^ As this study included both inpatients and outpatients, the true frequency of PEF cases may have been higher because not all fever episodes may have been reported. However, in another prospective study tracking adverse events in children within 72 h post‐endoscopy, 61 fever episodes were recognised in 9577 procedures performed, equalling also a PEF rate of 0.6%. A slightly higher rate of fever (2%) after upper GI endoscopy in children was reported in a telephonic interview study by Ammar et al.^11^

Therefore, our study suggests that paediatric patients with IF are at an increased risk of developing PEF. The pathophysiology of PEF is not yet fully understood. Analogous to postoperative fever, the contributing factors in focus are inflammation from tissue injury/mucosal tears, drug‐induced fever, physiological periinterventional stress and bacterial translocation.^10^, ^12^ In this regard, it can be speculated that patients with IF, who are prone to specific risk factors including mucosal barrier dysfunction, intestinal bacterial overgrowth and mucosal inflammation, are at higher risk for endogenous translocation.^13^, ^14^, ^15^, ^16^, ^17^, ^18^ In this context, we observed a mild increase in C‐reactive protein in six patients with PEF (measured in *n* = 7) and a negative peripheral blood culture in those who were analysed (*n* = 2). Nevertheless, microbiology and inflammatory markers were evaluated in only a limited number of patients and not in a systematic manner, preventing any definitive conclusions.

Transient bacteraemia occurs with many activities of daily living and procedures, especially dental procedures (30%–90%).^19^ Reported rates of bacteraemia are up to 8% following upper GI endoscopy, up to 25% after sigmoidoscopy and colonoscopy, and the risk can be increased in interventional procedures such as stricture dilatation.^20^ Bacteraemia as a consequence of translocation of endogenous bacterial flora is often asymptomatic, self‐limiting and rarely leads to clinical infections in immunocompetent hosts.^6^, ^21^

The North American Society for Pediatric Gastroenterology, Hepatology, and Nutrition endoscopy committee recommends considering antibiotic prophylaxis for sepsis prevention only in children undergoing endoscopy with a presumably high rate of bacteraemia (dilatation, sclerotherapy, variceal band ligation, and endoscopic retrograde cholangiopancreatography).^22^

However, there is a lack of data in the unique group of paediatric patients with IF and a long‐term CVC. In a questionnaire study among 15 academic, paediatric gastroenterology centres by Snyder et al., 20% and 40% of centres responded to use AMP in children with CVC undergoing upper GI endoscopy and colonoscopy, respectively.^8^

Therefore, it is important to highlight that no clinical infection, including CLABSI, occurred in our study, encompassing more than 200 GI endoscopies. In addition, according to our analysis, i.v. AMP did not affect the PEF frequency. In 16% of the cases, an oral/enteral antibiotic regimen was in place as part of the intestinal bacterial overgrowth treatment. However, there was no significant difference between the two groups.

This finding is also important from an individual and health policy‐related perspective: the restrictive use of antibiotics could save resources, avoid allergic reactions, and decrease the burden of global antimicrobial resistance and selective pressure on resistant intestinal pathogens in individual patients.

We had the unique opportunity to compare children with i.v. AMP versus those without AMP because of an institutional policy change in 2022. Therefore, the decision to use or not use AMP was not based on the individual patient's risk factors or comorbidities. Notably, none of the patients was immunocompromised.

Our study was limited by its single‐centre retrospective design and unequal group sizes, with 71% of patients receiving i.v. AMP. However, more than 200 endoscopies were analysed, and the strength of the study is that only inpatients were included and accurate detection of PEF was ensured, minimising the risk of under‐ or overreporting. No blood cultures were drawn; therefore, it remains speculative whether the increased PEF frequency in children with IF could be caused by an increase in endogenous bacterial translocation. Timely immunological response could have cleared bacteraemia; however, it is more likely that—in analogy to postoperative fever—tissue damage and periinterventional stress with rise in cytokine plasma concentrations contribute to the development of PEF.^10^

## 5 CONCLUSION

Children with IF are at risk of developing PEF. However, no clinical infections occurred, suggesting that PEF was caused by a self‐limiting process induced by an unspecific acute phase reaction rather than bacteraemia. This finding was supported by the fact that AMP does not prevent PEF. Therefore, routine administration of peri‐interventional i.v. AMP to reduce the risk of endoscopy‐related infectious complications in otherwise healthy, immunocompetent children with IF and CVC may not be indicated. These findings may have implications for clinical practice and guideline development. Further prospective studies are required to confirm these observations.

## CONFLICT OF INTEREST STATEMENT

The authors declare no conflicts of interest.
